# Cutaneous Carcinosarcoma: A Small Case Series and Review of the Literature of a Rare Skin Tumor

**DOI:** 10.7759/cureus.9569

**Published:** 2020-08-05

**Authors:** Ethan Y Song, Sean J Wallace, Hina Sheikh, Randolph Wojcik Jr., Chetan Nayak

**Affiliations:** 1 Plastic and Reconstructive Surgery, University of South Florida Morsani College of Medicine, Tampa, USA; 2 Division of Plastic & Reconstructive Surgery, Lehigh Valley Health Network, Allentown, USA; 3 Pathology, Health Network Laboratories, Allentown, USA; 4 Otolaryngology, Lehigh Valley Health Network, Allentown, USA

**Keywords:** cutaneous carcinosarcoma, sarcomatoid carcinoma, skin cancer

## Abstract

Carcinosarcomas are composed of epithelial and mesenchymal elements and primarily present within visceral organs. Despite being potentially aggressive, they are a rare diagnosis in the skin, and few manifestations have been reported to date. In this report, we describe two separate cases of carcinosarcoma presenting as nonhealing scalp wounds. Patient A: a 57-year-old male with a nonhealing skin lesion of ten years successfully treated with wide-local excision and local ortichochea flap reconstruction. Patient B: a 75-year-old female that presented with a painless, slow-growing hemorrhagic mass of 7 years invading the skull and dura ultimately requiring craniectomy and free-tissue transfer with anterolateral thigh flap. Cutaneous carcinosarcomas have more favorable outcomes due to low metastatic rates likely due to earlier detection, but delayed presentation can be fatal. Histopathological analysis is critical for determining diagnosis and prognosis. Adequate reconstruction after wide base excision varies and follows the reconstructive ladder/elevator ranging from primary closure up through free-tissue transfer. With cutaneous manifestations of carcinosarcoma seldom reported in the literature, it is our hope that reporting unusual instances such as this will raise awareness and allow for earlier diagnoses, treatments, and reconstructions.

## Introduction

Cutaneous carcinosarcoma is a rare, biphasic cancer composed of epithelial and mesenchymal tissues that were first described in 1972 [1]. Carcinosarcomas have been primarily associated with visceral organs and previously described most commonly in the uterus, ovary, lung, gastrointestinal tract, prostate, thyroid, and testis [2-4]. Secondary to their late presentations viscerally, they tend to have high malignant potential and poor prognoses. It is not feasible to distinguish cutaneous carcinosarcoma from other similar-appearing skin cancers based on clinical appearance alone. Diagnosis requires clinical suspicion and subsequent histopathologic sampling of concerning tissue. Histomorphologic biphasic cell populations of the epithelial and mesenchymal components showing differential immunoreactivity with epithelial element being positive for keratin markers and p63 while mesenchymal elements showing reactivity for markers such as CD10, CD99 and variable (weaker to absent) epithelial marker expression are defining diagnostic features [5]. Negative prognostic factors include aggressive histologic subtypes such as squamous cell component, earlier age of presentation, tumor size >2 cm, and nodal involvement [6,7].

In this report, we describe two unusual cases of carcinosarcoma presenting on the scalp. We present the clinical, surgical, and histopathological features, discuss the treatment and reconstructive methods based on tissue deficit, and finally review the published medical literature on non-visceral, cutaneous carcinosarcomas.

## Case presentation

Patient A

A 57-year-old male presented with a painless mass at the scalp vertex that had been present for 10 years’ time. Past medical history was most significant for BRCA2 mutation, squamous cell carcinoma (SCC) of the eyelid status-post excision and radiation therapy, and prostate cancer status-post radical prostatectomy and radiation therapy. Family history was significant for cancers including lymphoma, prostate, pancreatic, skin, ovarian, breast, and thyroid.

**Figure 1:**
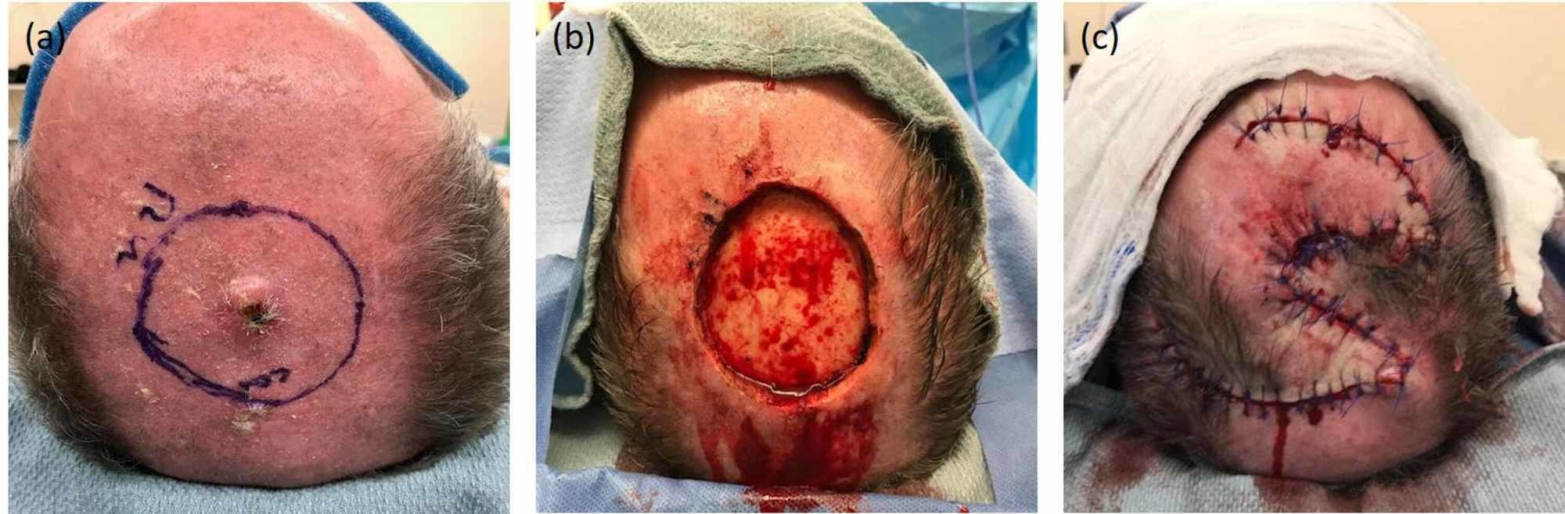
Patient A: (A) pre-excision, (B) post-excision defect illustrating exposed bone devoid of pericranium, and (C) reconstructed scalp wound with local tissue rearrangement (ortichochea flap)

**Figure 2:**
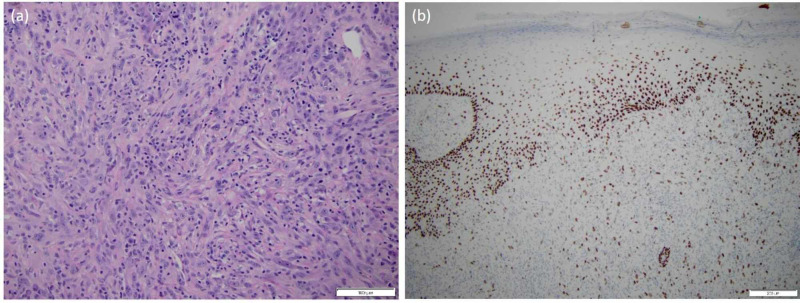
Patient A: (A) a representative routine H&E section at 20x showing spindled and undifferentiated histomorphology classically seen in carcinosarcoma and (B) a representative immunohistochemical stain at 10x showing variable p63 positivity targeting the diagnosis of carcinosarcoma H&E, hematoxylin and eosin

On exam, he had a palpable, tender, non-mobile 2 x 2 cm cutaneous mass at scalp vertex (Figure 1A). A shave biopsy revealed biphasic cytology: one component with moderate-to-poorly differentiated squamous carcinoma, connected to and emanating from the epidermis, and the other more pleomorphic undifferentiated spindled and histiocytoid cells with atypical mitotic figures, representing a sarcomatous component. The immunoprofile mirrored morphologic differences in these populations (carcinomatous areas were strongly positive for keratins, CK5/6 and AE1/AE3 and also p63; sarcomatous areas were strongly positive for CD68, CD163 and show only scattered staining of single cells with AE1/AE3). Based on histologic and immunohistochemical observations, the tumor was determined to be a carcinosarcoma (Figure 2A, B).

Radiological evaluation was negative for the involvement of the cervical or suboccipital lymph nodes. He underwent a wide local excision with frozen section. The surgical defect measured 5.5 x 5.5 cm and had exposed skull devoid of pericranium (Figure 1B). To provide adequate and durable well-vascularized soft tissue coverage, an ortichochea local tissue rearrangement was designed utilizing three separate rotational flaps equally positioned around the defect (Figure 1C). Final pathology revealed carcinosarcoma with negative margins.

At outpatient follow-up through 12 months, he had no evidence of recurrence, metastasis, or wound-healing complications.

Patient B

A 75-year old female with no significant past medical history presented for evaluation of a painless, non-healing wound on her scalp of seven years’ time. Associated symptoms included recent and rapid significant unexplained weight loss, as well as anemia.

**Figure 3:**
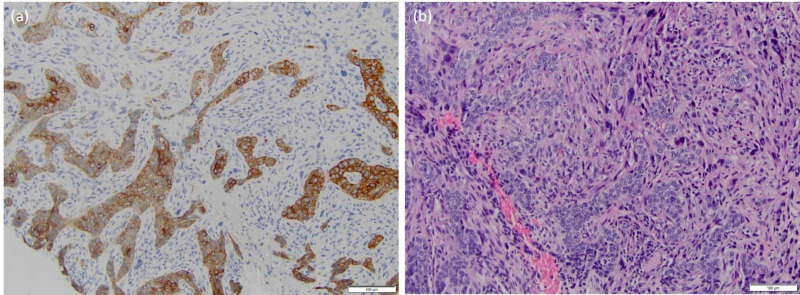
Patient B: (A) basal cell carcinosarcoma showing biphasic cytology at 20x and (B) differential cytokeratin immunostaining at 20x magnification with sarcomatoid cells staining negative

On examination, she had a 7 x 6 cm ulcerating, fungating mass at the scalp occiput extending towards the right parietal region, as well as a palpable lymph node along the anterior border of the right sternocleidomastoid muscle. A punch biopsy revealed mixed basal cell carcinoma (BCC) along with a poorly-differentiated malignant component of spindled, epithelioid, and pleomorphic cytology closely intermingled with the BCC cells. On immunostains, CD99 showed extensive positivity of BCC and poorly differentiated tumor cells. These poorly differentiated cells were positive for vimentin, showed focal staining for smooth muscle actin, and were negative for cytokeratin AE1/AE3, cytokeratin 903, S100, MART and CD45. Poorly differentiated malignant cells were negative for HMB45, CD3, CD20, CD34, synaptophysin and cytokeratin 20, further excluding the possibility of co-existent melanoma, lymphoma, angiosarcoma and Merkel cell carcinoma. The histopathologic morphology and immunoprofile supported a diagnosis of basal cell carcinosarcoma (Figure 3A, B).

**Figure 4:**
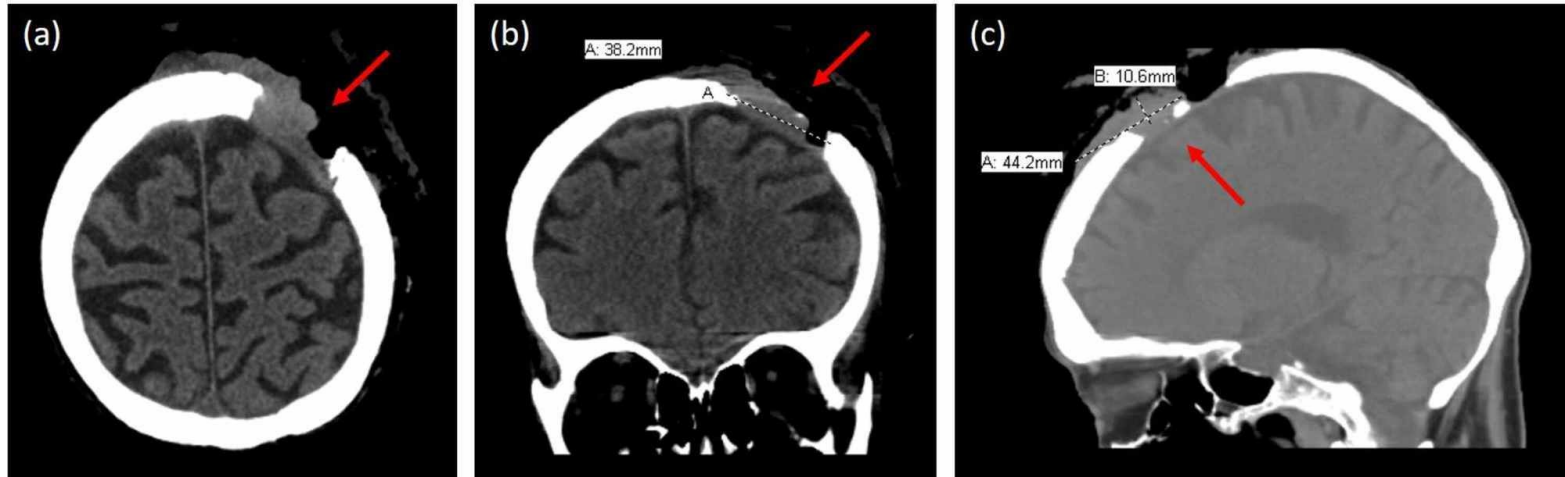
Patient B: Computed tomography scan in the (A) axial, (B) coronal and (C) sagittal views illustrating cranial bone and brain involvement of carcinosarcoma

CT of the head illustrated an underlying 3.7 x 3.8 cm component involving skull and dura (Figure 4A-C). She underwent pre-operative chemotherapy with Vismodegib. At surgical extirpation, a 7 cm hemorrhagic tumor was removed. The defect measured 12 x 7 cm. Within the wound bed, there was skull devoid of pericranium, as well as a transcranial defect with exposed brain. To provide adequate and durable well-vascularized soft tissue coverage, an anterior lateral thigh free-tissue transfer was performed. Final pathology confirmed carcinosarcoma with negative margins.

Initial outpatient follow-up revealed her to be recovering well; however, within 30 days, she was hospitalized due to a fall and was found to have a recurrence of the carcinosarcoma with extension into the brain on trauma imaging. She underwent a second palliative surgery and received postoperative radiation therapy, yet her condition continued to deteriorate, and she ultimately succumbed to her disease.

## Discussion

Rarely reported as a primary tumor of the skin with only around 130 total cases documented, carcinosarcomas have been associated with better outcomes secondary to earlier diagnosis. Worldwide, few cases of cutaneous carcinosarcoma have been reported in the medical literature. A review of PubMed Medline involving reports of carcinosarcoma utilizing search terms (cutaneous carcinosarcoma [Title/abstract] AND carcinosarcoma of the skin [Title/abstract]) AND ((skin OR subcutaneous OR cutaneous OR superficial)) revealed only 65 articles, with the majority as case reports and/or small case series (Table 1).

**Table 1:** Review of PubMed Medline literature involving case reports and case series of non-visceral carcinosarcomas

PMID	Year	Article
30618513	2019	Cutaneous Carcinosarcoma: a Clinicopathologic and Immunohistochemical Analysis of 11 Korean Cases
30789518	2019	Cutaneous Carcinosarcoma of the Medial Canthus Discovered on Mohs Debulk Analysis
29666695	2018	Primary Cutaneous Carcinosarcoma: A cutaneous neoplasm with an exceptional presentation.
29954591	2018	Pilomatrical Carcinosarcoma: Report of a Case with Comparative Genomic Hybridizsation Analysis
30283313	2018	A Case of Primary Cutaneous Basal Cell Carcinoma
29954591	2018	Trichoblastic Carcinosarcoma of the skin: A Case Report and Literature Review
29624700	2018	Sarcomatoid Pilomatrix Carcinoma
29484002	2018	Giant Pendulous Carcinosarcoma - Squamous cell Carcinoma Type - of the Leg - A Case Report and Review of the Literature
29293124	2018	Cutaneous Metaplastic Carcinoma: Report of a Case With Sebaceous Differentiation
28481030	2017	Pilomatrical Carcinosarcoma of the Cheek: Immunohistochemica and Molecular Analysis of Beta-Catenin
28452720	2017	Carcinosarcoma of Skin (sarcomatoid carcinoma) - A Rare Non-Melanoma Skin Cancer (Case Review)
27730657	2017	Cutaneous Carcinosarcoma: A Series of Six Cases and a Review of the Literature
27910277	2016	Aggressive Growth of an Incompletely Excised Primary Cutaneous Basal Cell Carcinosarcoma on the Scalp: A case Report
27205905	2016	A Growing Scalp Nodule
27194674	2016	Trichoblastic Carcinosarcoma: An Authentic Cutaneous Carcinosarcoma
26272477	2016	Squamous cell carcinoma with osteoclast-like giant cells: a morphologically heterologous group including carcinosarcoma and squamous cell carcinoma with stromal changes
27163927	2016	Exophytic Scalp Tumor
25775619	2015	Primary Cutaneous Carcinosarcoma of the Basal Cell Subtype Should be Treated as High-Risk BCC
26268472	2015	Cutaneous Basal Cell Carcinosarcoma: Case Report and Literature Review
25328737	2014	Cutaneous Carcinosarcoma with Metastasis to the Parotid Gland
24978645	2014	Cutaneous Carcinosarcoma Arising from a Burn Scar
24698934	2014	Basal Cell Carcinosarcoma: A Report of 4 Cases and Review of the Literature
24343211	2014	Carcinosarcoma: A Primary Cutaneous Tumor with Biphasic Differentiation
24335643	2014	Basal Cell Carcinosarcoma with PTCH1 Mutations in both Epithelial and Sarcomatoid Primary Tumor Components and in the Sarcomatoid metastasis
23205622	2013	Primary Cutaneous Carcinosarcoma of the Shoulder: Case Report with Literature Review
22960838	2012	Primary Cutaneous Sarcomatoid Carcinoma
22439885	2012	Sarcomatoid Carcinoma of the Hand: A Clinical Case with an Aggressive and Uncommon Presentation
21658982	2012	Merkel Cell Carcinosarcoma: Merkel Cell Carcinoma with Embyronal Rhabdomyosarcoma-like Component
21788565	2011	Carcinosarcoma Derived from Nevus Sebaceus
21464722	2011	Carcinosarcoma Ex Eccrine Spiradenoma of the Vulva: Report of the First Case
20598063	2010	Carcinosarcoma of the Vulva: A Case Report
20570108	2010	Metastatic Cutaneous Carcinosarcoma to the Tongue
20148837	2010	Primary Cutaneous Carcinosarcoma: Dermatoscopic and Immunohistochemical Features
22282673	2010	Primary Carcinosarcoma of the Skin
19684512	2009	Cutaneous Carcinosarcoma with Myoepothelial Differentiation: Immunohistochemical and Cytogenetic Analysis of a Case Presenting in an Unusual location
18793937	2008	Basal Cell Carcinoma with a Sarcomatous Component (carcinosarcoma): a series of 5 cases and review of the literature
18412870	2008	Primary Cutaneous Carcinosarcoma: Case Report with Expanded Immunohistochemical Analysis
18261109	2008	Cutaneous Carcinosarcoma: A Report of a Case with Myofibroblastic Sarcomatous Component
18212548	2008	High Grade Trichoblastic Carcinosarcoma
18482037	2007	Cutaneous Carcinosarcoma Comprising Basal Cell Carcinoma and CD34+ Fibrosarcoma
17113530	2006	Are Primary Cutaneous Carcinosarcomas Underdiagnosed? Five Cases and a Review of the Literature
16919037	2006	Cutaneous Carcinosarcoma - Basal Cell Carcinoma with Osteosarcoma
16733452	2006	Carcinosarcoma of the Skin
16381134	2005	Primary Carcinosarcoma of the Helix of the Ear
16176303	2005	Primary Cutaneous Carcinosarcoma Arising in a Patient with Nevoid Basal Cell Carcinoma Syndrome
15943184	2005	Case Report and Literature Review: Primary Cutaneous Carcinosarcoma
15858510	2005	Primary Carcinosarcoma of the Skin
15677972	2005	Carcinosarcoma Arising in a Patient with Multiple Cylindromas
15606673	2005	Biphasic Sarcomatoid Basal Cell Carcinoma (Carcinosarcoma): Four Cases with Immunohistochemistry and Review of the Literature
15491328	2004	Primary Sweat Gland Carcinosarcoma of the Scrotal Skin
15249861	2004	Low-grade Trichobastic Carcinosarcoma of the Skin
12928545	2003	Basal Cell Carcinoma with Sarcomatoid Features (sarcomatoid carcinoma): Report of a Case and Review of the Literature
12834489	2003	Monophasic Sarcomatoid Carcinoma of the Scalp: A Case Mimicking Inflammatory Myofibroblastic Tumor and a Review of Cutaneous Spindle Cell Tumors with Myofibroblastic Differentiation
12602972	2003	A Case of Sarcomatoid Carcinoma of the Skin
12410152	2002	Carcinosarcoma of the Scalp
10469102	1999	Primary Carcinosarcoma of the Skin: Report of a Case and Review of the Literature
9261471	1997	Primary Metastatic Carcinoma (Carcinosarcoma) of the Skin: A Clinicopathologic Study of Four Cases and Review of the Literature
8989936	1996	Carcinosarcoma of the Skin. Case Report and Literature Review
8639057	1996	Carcinosarcoma Arising in Eccrine Spiradenoma of the Breast. Report of a Case and Review of the Literature
7541771	1995	Carcinosarcoma of Skin
8366217	1993	Carcinosarcoma of the Skin: Immunohistochemical and Electron Microscopic Observations
3280629	1988	Carcinosarcoma of the Skin
3223796	1988	Carcinosarcomas of the Skin - Report of Two Cases
7309937	1981	Carcinosarcoma of the Skin. Case Report and Review
5073912	1972	Carcino-Sarcoma of the Skin

Cutaneous carcinosarcoma tend to be found in adults in chronically sun-exposed regions after the fifth decade of life with a male predominance 1.7:1 [5,8,9]. These tumors are known for their biphasic nature, rapid rate of growth, ulceration, and exophytic-appearance that can be misleading to even the most-experienced clinicians. Several histologic forms of carcinosarcoma of the skin have been described, including basal cell, squamous cell, pilomatrical, and trichoblastic carcinosarcoma [10-12]. As opposed to adnexally derived carcinosarcomas, epidermally derived carcinosarcomas are reported to have a better five-year survival rates, with disease-free survival of 70% compared to 25%, respectively [6]. However, the survival rates of cutaneous carcinosarcomas are not as high as that of conventional BCC and SCC, of which five-year survival rates can reach over 95% [13].

There are currently several existing hypotheses to explain the development of carcinosarcomas, which include divergent differentiation of a single progenitor cell into malignant epithelial and mesenchymal populations (monoclonal hypothesis), origin from two separate progenitor cells (multiclonal hypothesis), and a collision tumor of two unique neoplasms [6,14]. At present, the monoclonal hypothesis is the most accepted, with a study by Bigby et al. reporting that sarcomatous components have been recognized as a metaplastic carcinoma of epithelial components [7].

Since carcinosarcomas are a biphasic malignancy, the epithelial components can be a mix of BCC due to mutations in PTCH1, p53, p63, and p13 gene mutations and SCC from point mutations in p53 [5]. The sarcomatous component is now accepted to be the result of divergent mesenchymal differentiation, or metaplasia, of the epithelial component [14]. The mesenchymal constituents have been described as either homologous (site-appropriate) or heterologous (site-inappropriate). Homologous forms include leiomyosarcoma and pleomorphic sarcoma, while heterologous forms include osteosarcoma, chondrosarcoma, and rhabdomyosarcoma [3]. More than one subtype can coexist in the same tumor.

To add to existing literature, we report two cases of carcinosarcoma of the scalp. Interestingly, both patients had nonhealing scalp masses secondary to trauma. Surgical pathology of Patient A demonstrated biphasic cytology with moderate to poorly differentiated squamous carcinoma connected to the epidermis and a pleomorphic spindled and histiocytoid cell population significant for sarcomatous component. The sarcomatous areas were strongly positive for CD68 and CD163. IHC was positive for keratins, CK5/6, AE1/AE3, as well as p63 in the epithelioid component with variable weaker positivity p63 in sarcomatoid cells. This is in agreement with past reports that highlight co-expression of p63 in epithelial and spindle cells [5]. Similarly, surgical pathology for Patient B showed IHC positive vimentin and smooth muscle actin and complete negativity for cytokeratins and p63, suggesting a more complete sarcomatous transformation. The loss of epithelial phenotype including cytokeratin AE1/AE3 and replacement by pleomorphic or spindle morphology accompanied by gain of vimentin expression has been previously described by Brasanac et al. [15].

Interestingly, the type and ratio of sarcomatous elements do not influence prognosis; rather, survival may depend more on the epithelial component. When the squamous cell component concentrations are greater, metastatic rates have been reportedly higher (12%-50%) when compared to basal cell components (2%) [5,6,16]. Of the 47 cases analyzed in a report by Zbacnik et al., there was only one metastatic case of axillary basal cell carcinosarcoma to the lung. Furthermore, a recent study of 11 cutaneous carcinosarcomas by Kwak et al reported three cases of metastases to local lymph nodes, two of which were SCC and one keratoacanthoma; however, they did not identify a significant relationship between primary tumor location or size due to a limited sample [17]. Based on these observations, in our report, Patient A had a more lethal form of carcinosarcoma due to the squamous cell component compared to Patient B with a basaloid component. The outcomes of the two patients in our series were contrary to general prognostic factors, as Patient B presented earlier with a less aggressive histologic form of carcinoma and was without distant metastases, but ultimately succumbed to her disease. Along with Bourgeault et al., we agree that a cutaneous carcinosarcoma of the basaloid subtypes should be treated as a high-risk basal cell carcinoma [18]. In both cases, our patients presented later in the disease stage in part due to the negligence of nonhealing wounds, further emphasizing the need for increased awareness of such conditions.

As previously reported, carcinosarcomas may present in various shapes and sizes; therefore, management depends on clinical and histological features [8]. There has been no consensus on definitive margins for excision for cutaneous primary manifestations, as reports describe a range from 0.5-3.5 cm margins [5,9,19,20]. As these defects can be large and in a prominently visible location, it is important for dermatologists to consider referral to plastic surgery for adequate reconstruction. Reconstruction follows traditional teaching in plastic and reconstructive surgery utilizing the reconstructive ladder and/or elevator, ranging from healing by secondary intent up through free-tissue transfer. Depending on the location, size of the defect, and extent of devoid tissue and type, an adequate reconstruction can be as important as the cancer resection to protect any vital structures that may have been exposed. In Patient A, bone deficient of pericranium was exposed in the 5 x 5 cm defect. Therefore, a well-vascularized soft tissue rearrangement in the form of an ortichochea flap was an appropriate reconstruction. In Patient B, the wound was significantly larger (12 x 7 cm) and extended transcranially, thus exposing bone denuded of pericranium and brain lacking meninges. In this instance, no local options were available to fill the defect and the most appropriate reconstruction included utilizing a thin and pliable anterior lateral thigh free-tissue transfer.

Cutaneous carcinosarcomas may be underreported due to lack of awareness, from both a patient and clinician perspective, and can ultimately prove fatal [16]. Fortunately, unlike skin adnexal and visceral carcinosarcomas, epidermal derived carcinosarcomas may be more visible leading to earlier detection, adequate management, and follow-up. Nevertheless, with cutaneous carcinosarcoma seldom reported in the literature, it is our hope that sharing our experience will not only add to the database of published literature, but also raise awareness of extra-visceral manifestations, and allow for earlier diagnosis and treatment.

## Conclusions

Compared to visceral counterparts, cutaneous carcinosarcomas have more favorable outcomes due to low metastatic rates likely due to earlier detection, but delayed presentation can be fatal. Nonhealing wounds of the scalp should be properly investigated for potential underlying malignancy. With cutaneous manifestations of carcinosarcoma seldom reported in the literature, it is our hope that reporting unusual instances such as this will raise awareness and allow for earlier diagnoses, treatments, and reconstructions.
